# Typology of patients with fibromyalgia: cluster analysis of duloxetine study patients

**DOI:** 10.1186/1471-2474-15-450

**Published:** 2014-12-23

**Authors:** Ilya A Lipkovich, Ernest H Choy, Peter Van Wambeke, Walter Deberdt, Doron Sagman

**Affiliations:** Quintiles, 4820 Emperor Boulevard, Durham, NC 27703 USA; Section of Rheumatology, Institute of Infection and Immunity, Cardiff University, Tenovus Building, Heath Park Campus, Cardiff, CF14 4XN UK; University Hospitals Leuven, Herestraat 49, B-3000 Leuven, Belgium; S.A. Eli Lilly Benelux N.V, Markiesstraat 1, B-1000 Brussel, Belgium; Eli Lilly and Company, 3650 Danforth Avenue, Toronto, Ontario M1N 2E8 Canada

**Keywords:** Fibromyalgia, Duloxetine, Cluster analysis, Patient subgroups, Prediction

## Abstract

**Background:**

To identify distinct groups of patients with fibromyalgia (FM) with respect to multiple outcome measures.

**Methods:**

Data from 631 duloxetine-treated women in 4 randomized, placebo-controlled trials were included in a cluster analysis based on outcomes after up to 12 weeks of treatment. Corresponding classification rules were constructed using a classification tree method. Probabilities for transitioning from baseline to Week 12 category were estimated for placebo and duloxetine patients (N_total_ = 1188) using logistic regression.

**Results:**

Five clusters were identified, from “worst” (high pain levels and severe mental/physical impairment) to “best” (low pain levels and nearly normal mental/physical function). For patients with moderate overall severity, mental and physical symptoms were less correlated, resulting in 2 distinct clusters based on these 2 symptom domains. Three key variables with threshold values were identified for classification of patients: Brief Pain Inventory (BPI) pain interference overall scores of <3.29 and <7.14, respectively, a Fibromyalgia Impact Questionnaire (FIQ) interference with work score of <2, and an FIQ depression score of ≥5. Patient characteristics and frequencies per baseline category were similar between treatments; >80% of patients were in the 3 worst categories. Duloxetine patients were significantly more likely to improve after 12 weeks than placebo patients. A sustained effect was seen with continued duloxetine treatment.

**Conclusions:**

FM patients are heterogeneous and can be classified into distinct subgroups by simple descriptive rules derived from only 3 variables, which may guide individual patient management. Duloxetine showed higher improvement rates than placebo and had a sustained effect beyond 12 weeks.

**Electronic supplementary material:**

The online version of this article (doi:10.1186/1471-2474-15-450) contains supplementary material, which is available to authorized users.

## Background

Fibromyalgia (FM) has been defined by the American College of Rheumatology (ACR) as pain in all 4 quadrants and axial skeletal pain, together with at least 11 of 18 tender point sites (1990 ACR criteria [1]), although revised criteria are being proposed [2], and new preliminary diagnostic criteria for FM were published in 2010 [3]. About 2% of the US general population suffers from FM. Patients often also suffer from concomitant symptoms such as fatigue, headache, and sleep disturbance [3]. Mood disorders are also commonly diagnosed in these patients with approximately 25% to 40% of FM patients reporting symptoms of current major depressive disorder (MDD) [4, 5].

Fibromyalgia symptoms may be in part due to deficiencies in serotonin (5-HT) and norepinephrine (NE), 2 neurotransmitters that have been implicated in the mediation of endogenous analgesic mechanisms via the descending inhibitory pain pathways in the brain and spinal cord [6, 7]. Recent evidence suggests that in pathological pain states, these endogenous pain inhibitory mechanisms may be dysfunctional. An imbalance in these inhibitory mechanisms may contribute to the central sensitization and hyperexcitability of the spinal and supraspinal pain transmitting pathways, and may manifest as persistent/chronic pain [8]. These mechanisms of central sensitization and inhibition of the descending anti-nociceptive pathways due to a diminished availability of 5-HT and NE seem to play a key role in the pathogenesis of FM [9–11].

Duloxetine hydrochloride, hereafter referred to as duloxetine, is a potent, selective, and balanced serotonin and norepinephrine reuptake inhibitor (SNRI) in vitro and in vivo. It is approved by the Food and Drug Administration for the treatment of MDD [12, 13], generalized anxiety disorder [12, 14], FM [15, 16], diabetic peripheral neuropathic pain [17, 18], and chronic musculoskeletal pain [17]. With its dual reuptake activity achieved at the recommended dose of 60 mg/day (with minimal or no titration), duloxetine has a relatively early onset of action both in terms of pain and mood components of FM. Duloxetine’s analgesic effects in the supraspinal and descending inhibitory spinal pain pathways are essentially independent of its central antidepressant effects, explaining perhaps its therapeutic effect in distinct mood and pain syndromes as well as syndromes with considerable co-morbidity, such as FM.

In one Phase II study and three Phase III studies, patients with FM (with or without MDD) were effectively treated with duloxetine in various doses compared to placebo [4, 5, 16, 19]. An additional Phase III study evaluated the safety and tolerability of duloxetine during an extended period of up to 60 weeks and showed that long-term treatment is safe and well tolerated [20, 21].

Results in these studies have been described mainly in terms of average scores and mean changes on different symptom and functioning rating scales. Following on from previous studies that examined subgroups of FM patients [21, 22], and to obtain a better understanding of the frequency and extent of changes at an individual patient level, we used data from these registration studies in a *cluster* analysis to identify distinct subgroups of FM patients based on measures of pain, mental and physical impairment, global impression, and overall functioning. Descriptive rules were derived to classify patients into outcome categories in terms of their overall health status. The relationship between and relative importance of the pain, mood, and functioning domains in FM were also explored. Finally, changes in outcome categories over time were examined in an attempt to predict response after treatment with duloxetine, depending on patient characteristics at the start of treatment.

## Methods

### Data sets

Data from the 4 randomized, parallel, double-blind, placebo-controlled studies in FM, according to the 1990 ACR criteria [1], were included [4, 5, 16, 19]. These studies had similar inclusion and exclusion criteria and compared the effects of duloxetine against placebo in a total of 1,276 FM patients with or without MDD. Treatment duration varied between 12 and 58 weeks. Consequently, the last available score within the time interval from 2 to 12 weeks after baseline was used as outcome for the cluster analysis. Patients treated with duloxetine received doses of 30 mg once daily (QD) or twice daily (BID), 60 mg QD or BID, or 120 mg QD.

A fifth study in comparable FM patients [20], designed as a long-term safety study of duloxetine at doses of 60 and 120 mg QD, was used to assess long-term changes.

All evaluated studies were approved by the applicable Ethical Review Boards and conducted in line with Good Clinical Practice (according to International Conference on Harmonisation) and the Declaration of Helsinki. All patients provided their informed consent to participate in the study prior to any study procedures.

### Scales and assessments

Clusters were identified by measures of pain, mental and physical impairment (including fatigue), global impression, and overall functioning. A total of 18 outcome variables from the following scales were used:

- Pain: Brief Pain Inventory (BPI) (overall score for pain interference and average pain intensity) [23] and item 15 (pain) of the Fibromyalgia Impact Questionnaire (FIQ) [24].
- Mental impairment: mental composite summary score of the 36-item short form questionnaire (SF-36) [25], Beck Depression Inventory (BDI) total score [26], and FIQ items 19 (anxiety) and 20 (depression).
- Physical impairment: FIQ physical impairment subscore (average of FIQ items 1–11, multiplied by 3.33 to obtain a score between 0 and 10), FIQ items 14 (interference with work) and 16–18 (tiredness, awoke rested, and stiffness, respectively), and SF-36 physical composite summary score.
- Global impression: Patient Global Impression of Improvement (PGI-I) and the Clinician Global Impression of Severity (CGI-S) [27].
- Overall functioning: Sheehan Disability Scale (SDS) assessing global disability in 3 areas: work, social life, and family life [28].

BPI pain interference and average pain intensity, FIQ items, and the areas of the SDS are rated on an 11-point scale ranging from 0 (best) to 10 (worst). PGI-I and CGI-S are scored on a 7-point scale from 1 (best) to 7 (worst). BDI items are rated on a 4-point scale from 0 (best) to 3 (worst); the BDI total score ranges from 0 (best) to 63 (worst). Thus, higher scores indicate a more negative impact for these scales. The SF-36 mental and physical composite summary scores were derived by principal component analysis [29]; higher SF-36 scores indicate better health status or functioning (ranges: mental composite summary score from -1.3 [worst] to 80.7 [best]; physical composite summary score from 1.7 [worst] to 76.3 [best]).

Except for BDI, all scales were used in all 4 placebo-controlled studies. For the one study with no BDI data [5], BDI scores were estimated by linear regression analysis applied on Hamilton Depression Rating Scale (HAMD) items [30]. In the long-term safety study, all scales except for the SF-36 were employed.

### Statistical methods

#### Cluster analysis

In a first step, a cluster analysis was performed on all female patients to identify distinct groups of patients characterized by the outcomes described above after up to 12 weeks of duloxetine treatment. Following transformation of values into z-scores, a *k*-means clustering algorithm in JMP 6.0.2 software was applied to the data on the 18 outcome measures [31]. Clustering of multivariate data was visualized using a 2-dimensional *biplot* projection of patients and outcomes [32].

A heuristic approach was used to decide on the number of clusters, based on the reproducibility of clustering across different numbers of clusters. The stability (reproducibility) was evaluated by randomly dividing the dataset into 2 halves and applying the *k*-means procedure to both halves separately. The agreement between the two partitions was then evaluated using the adjusted Rand index [33]. The average index computed over 500 random data splits was plotted against the number of clusters to identify the number of clusters above which there was a substantial drop in reproducibility.

To confirm the outcome of this approach, an alternative procedure was applied, the theoretically validated JUMP method (available at http://www-rcf.usc.edu/~gareth/research/jump*, with documentation at*http://www-bcf.usc.edu/~gareth/research/jumpdoc.pdf) [34].

#### Identification and application of descriptive rules

In the next step, a classification tree algorithm was applied to the same data to derive descriptive rules that would allow the classification of a patient into outcome *categories* that mimic the identified *clusters*. The outcome variable was the class membership and the predictor variables were the same variables that were used in the clustering procedure. However, the SF-36 mental and physical composite summary scores were excluded so that these results could be projected on the long-term safety study in which the SF-36 was not employed. The algorithm, similar to the Classification and Regression Trees (CART) algorithm introduced by Breiman et al. in [35], sequentially splits data into 2 child subsets by choosing the best cut-off point from all available variables so as to make the resulting 2 subsets as “pure” as possible in terms of their class memberships. Specifically, a recursive partitioning algorithm available in the Partition platform of the JMP 6.0.2 software was used. The descriptive rules were then constructed for each outcome cluster by following the splits leading to the nodes of the tree that were most representative of that cluster.

The descriptive rules were then applied to all patients with available data, including patients treated with placebo, and at baseline and the 12-week endpoint.

#### Evaluation of probabilities for transitioning from baseline to endpoint categories

Shift tables for transitioning from baseline to endpoint categories were generated and frequencies compared between duloxetine and placebo. Switching probabilities were modeled in a multinomial logistic regression analysis that included categorical factors for baseline category, treatment, treatment by baseline category interaction and other baseline covariates (age, race, previous antidepressant use, body mass index [BMI], diagnosis of MDD) using SAS proc LOGISTIC. Stepwise variable selection was used to select relevant baseline covariates. The general form of the multinomial logistic model for *k* outcome categories with logit link function was used. This allowed estimation of the probability of switching from any baseline category to any specific outcome category, for a given treatment group and other covariates included in the logistic regression. The confidence intervals for the difference in switching probabilities across treatment groups and associated p-values were computed using proc NLMIXED.

#### Assessment of the long-term effect of duloxetine using categories

To assess the long-term effect of duloxetine, patients from the long-term safety study were categorized according to the descriptive rules identified in previous procedures using available data from Week 14 (LOCF interval: >Week 1 to Week 14) and Week 26 (LOCF interval: >Week 19 to Week 26). Similarly, as a supporting analysis, patient frequencies per category were computed for 2 of the 4 studies used in the cluster analysis that included an extension phase. A similar LOCF approach as for the long-term safety study was applied.

## Results

### Demographics and baseline characteristics

In the 4 studies included, a total of 1,253 patients had non-missing BPI and FIQ outcome scores after at least 2 weeks of treatment; 1,188 women and 65 men. Since the number of men was considered too low to draw reliable conclusions, only women were included in the analyses. Of these 1,188 women 724 were treated with duloxetine and 464 randomized to placebo. No relevant differences between treatment groups in demographics and baseline characteristics were seen at baseline. The overall mean (SD) age was 50.3 (11.0) years, 86.8% were Caucasian and 9.7% Hispanic, and mean (SD) BMI was 29.8 (7.0) kg/m^2^. A major depressive episode was present in 26.9% of patients and 47.4% had previously received antidepressants.

### Cluster analysis

**Figure 1:**
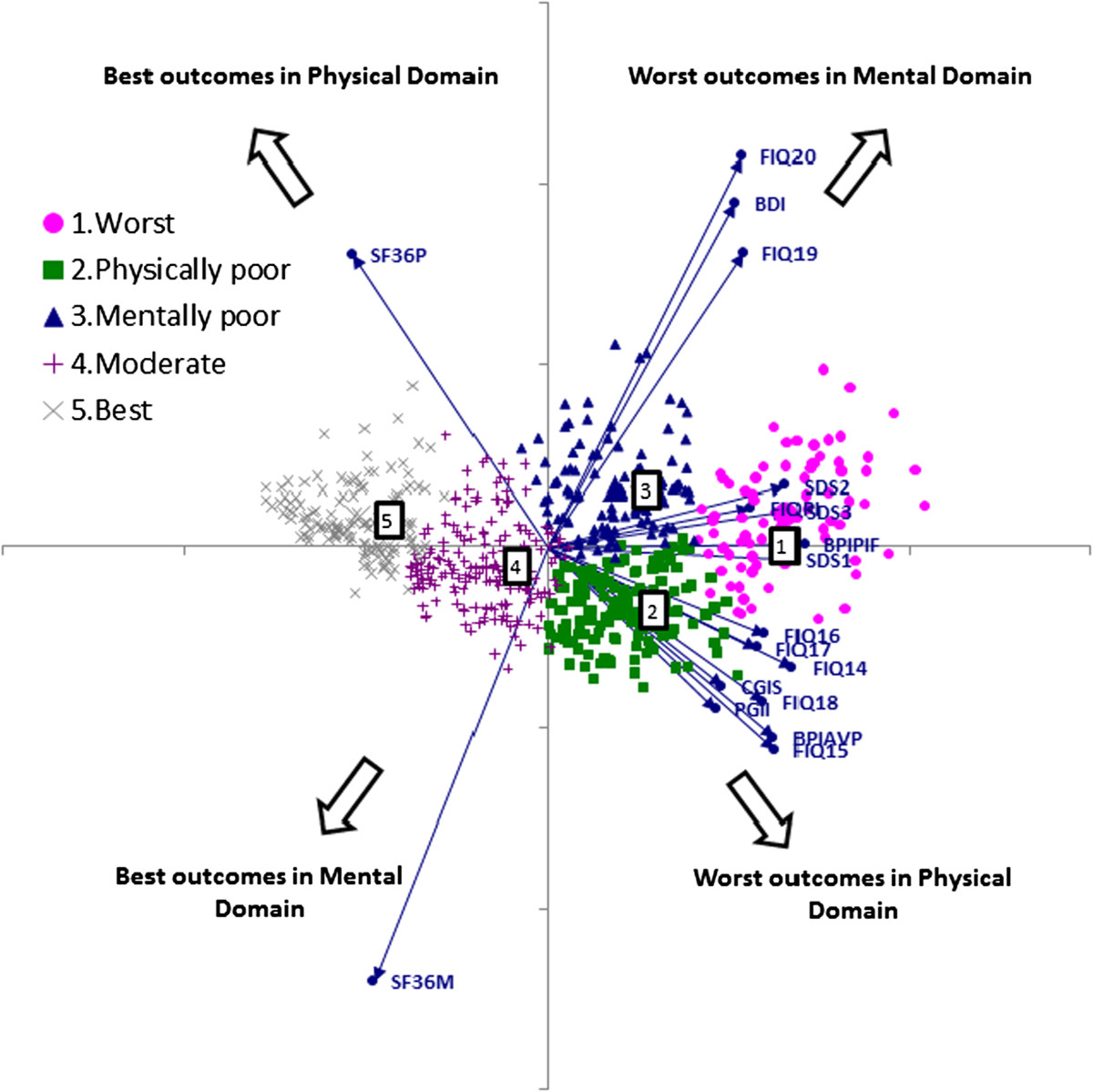
**Cluster analysis results projected on a biplot.** BDI = Beck Depression Inventory; BPIAVP = Brief Pain Inventory - average pain intensity; BPIPIF = Brief Pain Inventory - pain interference score; CGIS = Clinician Global Impression of Severity; FIQPI = Fibromyalgia Impact Questionnaire physical impairment; FIQx = Fibromyalgia Impact Questionnaire item x; PGII = Patient Global Impression of Improvement; SF36M = 36-item short form questionnaire – mental composite summary score; SF36P = 36-item short form questionnaire – physical composite summary score; SDSx = Sheehan Disability Scale domain x. Note: Symbols indicate patients, rays indicate outcome variables. Angles between rays reflect the degree of association between variables: variables that are highly related would be shown as co-directional rays (if positively correlated) or anti-directional (if negatively correlated); coordinates for patients are based on the first 2 principal components and, for the outcomes, on the loadings of individual variables on the principal components. Patients with similar outcomes are grouped together in the multivariate space which agrees with the class memberships from cluster analysis, indicated by color and symbol. Numbers in boxes indicate the centers of the respective cluster. The point at the intersection of the axes is associated with a hypothetical patient having average scores for all outcomes.

The initial cluster analysis was performed based on 631 duloxetine treated female patients who had non-missing scores for all 18 variables after at least 2 weeks of treatment. The *k*-means procedure with the number of clusters set as 4, 5, and 6 resulted in the selection of *k* = 5, leading to reasonably robust, i.e. reproducible, results. This number of clusters was also suggested by the JUMP procedure [34]. Furthermore, this choice of *k* turned out to be the most appropriate from a medical perspective with distinct characteristics per cluster. Table 1 presents the mean scores at Week 12 per identified cluster for each of the included scales and Figure 1 a projection graph (biplot) of the 5 clusters.

**Table 1:** **Mean scores and percent of patients meeting sds remission criterion by cluster at 12 weeks**

		Mean score				
Variable	Scale	Cluster 1 (worst),	Cluster 2 (physically poor),	Cluster 3 (mentally poor),	Cluster 4 (moderate),	Cluster 5 (best),
		N = 78	N = 135	N = 104	N = 185	N = 129
Pain						
BPI - Average pain intensity	BPIAVP	7.58	5.93	4.12	3.55	1.43
BPI - Pain interference (overall score)	BPIPIF	7.89	4.77	4.21	2.23	0.60
FIQ item 15: Pain	FIQ15	8.71	7.03	5.03	4.42	1.70
Mental impairment						
FIQ item 19: Anxiety	FIQ19	7.38	2.30	5.09	1.70	0.67
FIQ item 20: Depression	FIQ20	6.91	1.45	5.09	1.12	0.57
BDI total score	BDI	25.08	7.93	15.81	5.99	3.46
SF-36 mental composite summary score	SF36M	34.46	53.70	37.31	54.08	56.37
Physical impairment						
FIQ - Physical impairment	FIQPI	6.77	4.42	4.55	2.56	1.23
FIQ item 14: Interference with work	FIQ14	8.26	6.29	4.82	3.16	0.77
FIQ item 16: Tiredness	FIQ16	9.18	7.82	7.31	5.88	2.28
FIQ item 17: Awoke rested	FIQ17	9.19	7.66	7.08	5.66	2.46
FIQ item 18: Stiffness	FIQ18	8.79	6.87	5.69	4.58	1.88
SF-36 physical composite summary score	SF36P	24.88	24.51	32.77	34.13	45.90
Global impression						
CGI-S	CGIS	4.35	3.80	3.50	3.01	2.18
PGI-I	PGII	4.27	3.61	2.97	2.63	1.57
Overall functioning						
SDS - Work/school	SDS1	8.01	5.66	5.06	2.55	0.87
SDS - Social life	SDS2	7.64	5.26	5.36	2.07	0.73
SDS - Family	SDS3	7.82	5.59	5.33	2.38	0.83
SDS – Overall	SDS	23.5	16.5	15.7	7.00	2.43
SDS – Overall ≤6 (%) (remission criterion)		0.0	1.48	2.88	46.0	90.7

BDI = Beck Depression Inventory; BPI = Brief Pain Inventory; CGI-S = Clinician Global Impression of Severity; FIQ = Fibromyalgia Impact Questionnaire; N = Number of patients with available data; PGI-I = Patient Global Impression of Improvement; SF-36 = 36-item short form questionnaire; SDS = Sheehan Disability Scale.

Note: for SF-36 scores, the higher the score the better the patient’s well-being, while for all other scores, the higher the score the worse the patient’s well-being.

Based on these results, patients in Cluster 1 (“worst” cluster) are characterized by high pain levels and severe mental and physical impairment as shown by worst mean outcome scores in all examined areas (Table 1). Patients in Cluster 2 (“physically poor”) have high pain levels and high physical impairment, whereas patients in Cluster 3 (“mentally poor”) have moderate pain levels and high mental impairment. Patients in Cluster 4 (“moderate”) have considerably improved scores in all areas compared to Clusters 1–3 and are characterized by moderate levels of pain or mental/physical impairment. Mean scores for Cluster 5 (“best”) were further markedly improved, with low pain levels and nearly normal mental and physical status, reflecting a condition close to what one might expect in the general population. Outcome Clusters 1 and 3 had the highest proportion of patients with concomitant MDD at baseline – 53.9% and 39.4%, respectively; this proportion was lower in Cluster 5 (24.0%) and lowest in Clusters 2 (16.3%) and 4 (18.9%). The clusters can also be interpreted in terms of the proportion of patients meeting the Sheehan Disability Scale remission criterion (SDS overall ≤6). Consistent with general interpretation of the clusters, the percent of patients meeting the SDS remission criterion was very low (<3%) in Clusters 1, 2, and 3, but reached 46% in Cluster 4 and >90% in Cluster 5 (Table 1).

### Categories and descriptive rules

**Figure 2:**
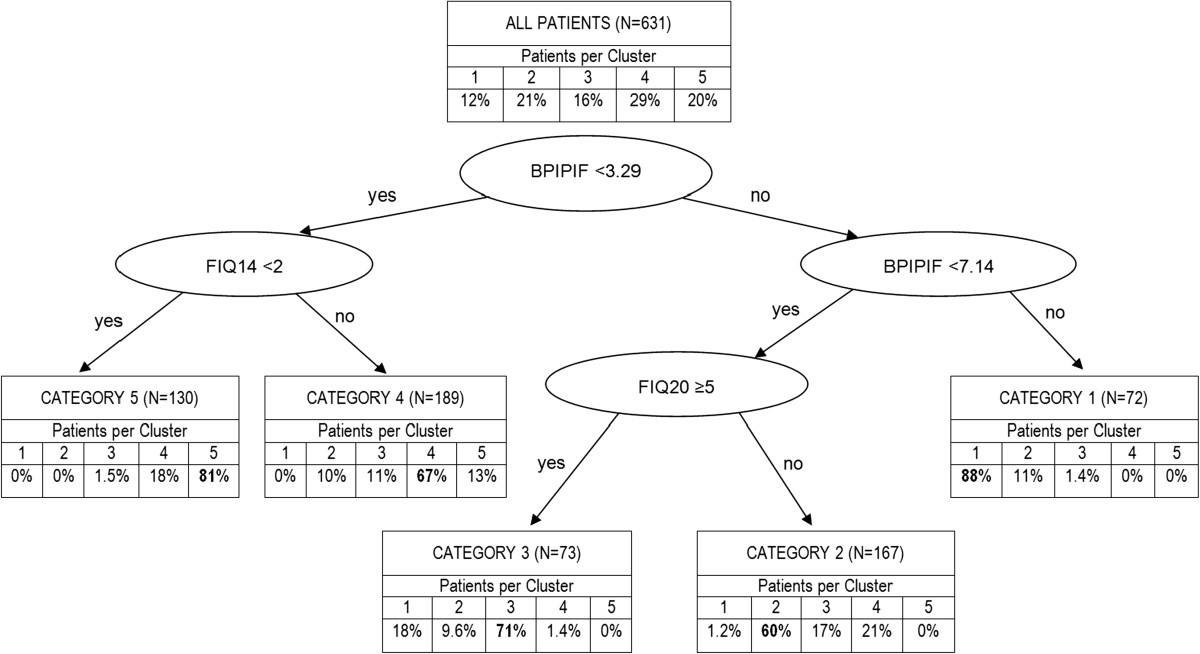
**Describing outcome clusters using a classification tree.** BPIPIF = Brief Pain Inventory (BPI) pain interference overall score; FIQ14 = Fibromyalgia Impact Questionnaire (FIQ) interference with work score; FIQ20 = FIQ depression score; N = Number of patients with available data who were also included in the cluster analysis.

In the next step, a classification tree recursive partitioning algorithm was applied on the same data in order to identify categories that mimic the clusters in terms of patient characteristics.As shown in Figure 2, the following 3 key variables and corresponding threshold values were identified: BPI pain interference overall scores (BPIPIF) of <3.29 and <7.14, respectively, an FIQ interference with work (FIQ14) score of <2, and an FIQ depression (FIQ20) score of ≥5. According to these variables and threshold values, simple descriptive rules for each category were defined allowing individual patients to be classified into any of the 5 categories. Within each resulting category, the majority of patients (60% to 88%) were from the corresponding cluster.

**Figure 3:**
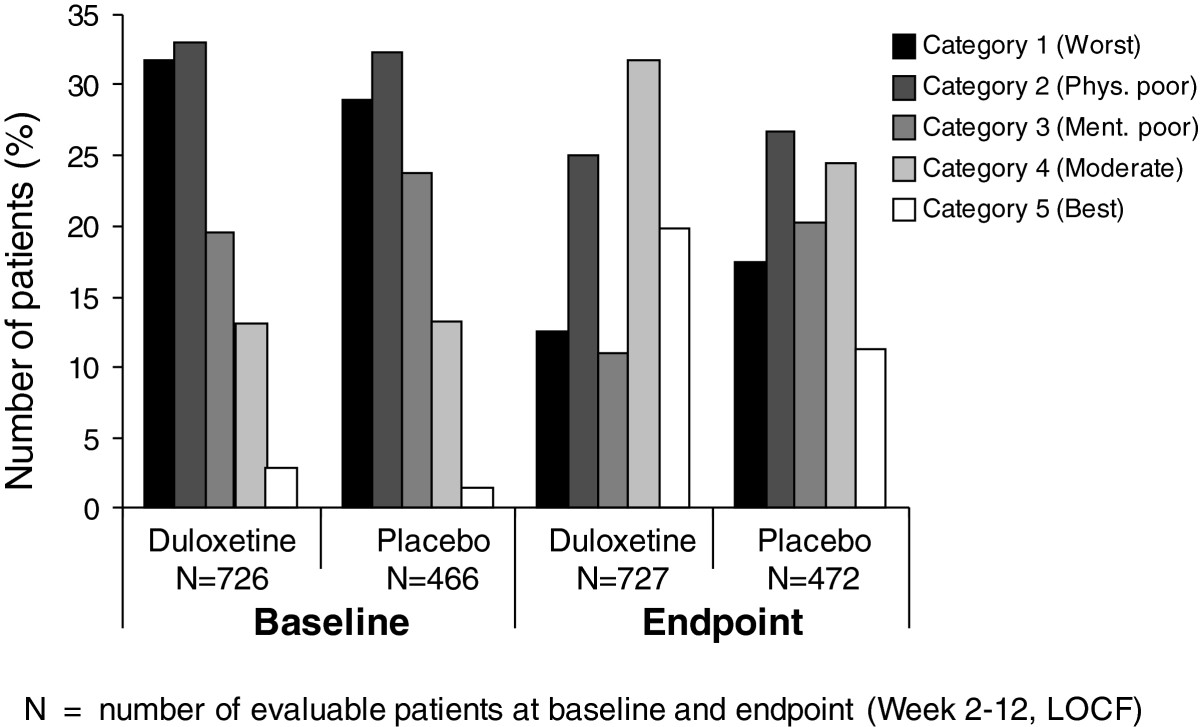
**Frequencies of patients per category at baseline and week 12.** Definition of categories, based on the classification rules from Figure 2: *Category 1*: BPIPIF ≥7.14; *Category 2*: 3.29 ≤ BPIPIF <7.14 AND FIQ20 < 5; *Category 3*: 3.29 ≤ BPIPIF <7.14 AND FIQ20 ≥ 5; *Category 4*: BPIPIF <3.29 AND FIQ14 ≥ 2, *Category 5*: BPIPIF <3.29 AND FIQ14 < 2. BPIPIF = BPI pain interference overall scores; FIQ = Fibromyalgia Impact Questionnaire; LOCF = last observation carried forward; N = Number of evaluable patients with outcome data at baseline and endpoint (Week 2–12, LOCF).

The accuracy of the approximation of the categories to the clusters was assessed with the area under the receiver operating characteristic (ROC) curve, which was at least 82% for each of the 5 clusters. This indicated a good approximation with reasonable accuracy despite the limited number of variables included.Figure 3 summarizes the descriptive rules that define the 5 outcome categories, and the respective classification of duloxetine and placebo group patients of the 4 studies at baseline and 12 weeks.Frequencies were similar between treatment groups at baseline. The majority of patients (>80%) at baseline belonged to the 3 worst categories 1 (worst), 2 (physically poor), and 3 (mentally poor), and only about 15% were in the 2 best categories. After 12 weeks of treatment, about half of patients in the duloxetine group (51.6%) compared to 35.6% of the placebo group were in the 2 best categories 4 (moderate) and 5 (best), with reductions in frequencies primarily observed in categories 1 and 3 (Figure 3). Specifically, the proportion of patients in these 2 categories reduced from 64.8% to 37.5% (-27.3%) in the duloxetine group and from 61.4% to 44.1% (-17.3%) in the placebo group.

### Probabilities for transitioning from baseline to week 12 category

**Figure 4:**
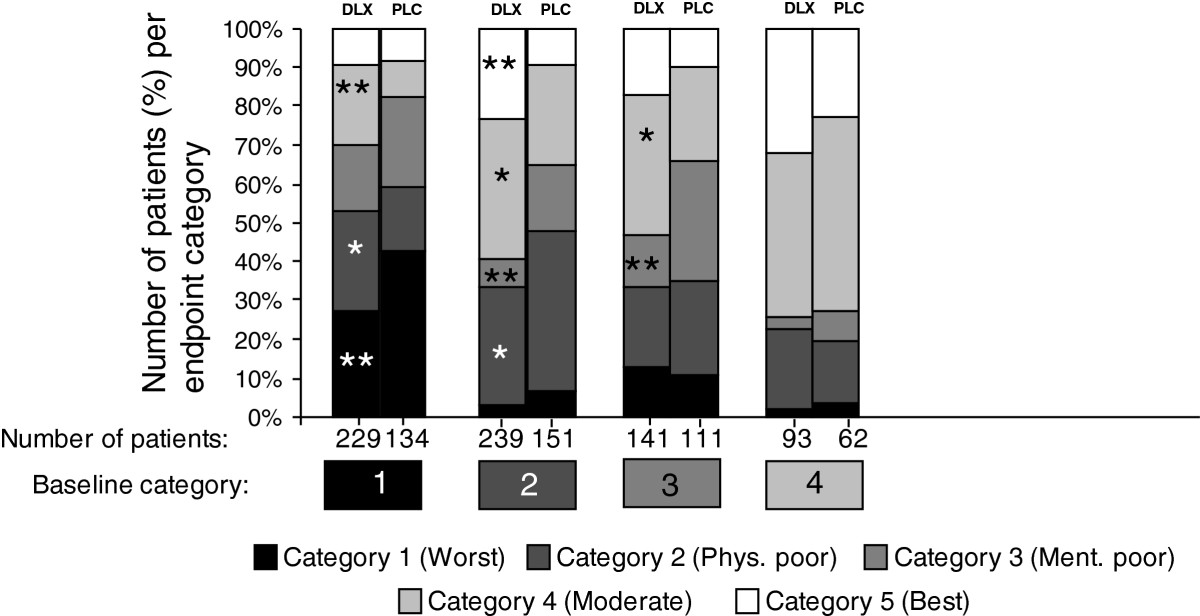
**Relative frequencies for transitioning from baseline to endpoint category.** The stacked bars show the distribution of patients from a given baseline category into 5 endpoint categories. The categories, based on the classification rules from Figure 2. (*Category* 1: BPIPIF ≥7.14; *Category 2*: 3.29 ≤ BPIPIF <7.14 AND FIQ20 < 5; *Category 3*: 3.29 ≤ BPIPIF <7.14 AND FIQ20 ≥ 5; *Category 4*: BPIPIF <3.29 AND FIQ14 ≥ 2, *Category 5*: BPIPIF <3.29 AND FIQ14 < 2). *p < 0.05, **p < 0.01 for the between-treatment difference in the probability of transitioning from a baseline category to the endpoint category. Note: Data from category 5 at baseline were excluded because only 20 duloxetine and 7 placebo patients fulfilled criteria for this category at baseline, which did not allow for any meaningful modeling and interpretation of transition probabilities.

Probabilities for changes in category from baseline to Week 12 were estimated using a logistic regression of the data set, excluding the few subjects with missing or best (5) baseline category or with missing outcome category (see Table 2 and Figure 4). For each of the poorer baseline categories, more patients treated with duloxetine than with placebo improved to a better category during the 12-week period. Whereas more than 10% of patients in either treatment group (12.8% and 10.8%, respectively) deteriorated from the “mentally poor” category to the “worst” category, only 2.9% and 6.0% of patients, respectively, in the “physically poor” category deteriorated to the “worst” category. For all but 1 category changes representing an improvement, frequencies were higher in the duloxetine group than in the placebo group (statistically significant for 5 such changes as indicated in Table 2 and Figure 4). For the change from category 2 (physically poor) to category 3 (mentally poor), the frequency was significantly higher in the placebo group than in the duloxetine group, which is due to more duloxetine patients having shifted from category 2 to categories 4 and 5 (Table 2 and Figure 4).

**Table 2:** **Relative frequencies for transitioning from baseline to endpoint category**

	Percentage of patients (duloxetine/placebo)					
	Endpoint category					
Baseline category	1 (worst)	2 (physically poor)	3 (mentally poor)	4 (moderate)	5 (best)	Total no. of patients
1 (worst)	27.5/42.5**	25.8/17.2*	16.6/22.4	21.0/9.7**	9.2/8.2	229/134
2 (physically poor)	2.9/6.0	30.5/41.7*	7.1/17.2**	36.4/25.8*	23.0/9.3**	239/151
3 (mentally poor)	12.8/10.8	20.6/24.3	13.5/30.6**	36.2/24.3*	17.0/9.9	141/111
4 (moderate)	2.2/3.2	20.4/16.1	3.2/8.1	41.9/50.0	32.3/22.6	93/62
Total no. of patients	90/80	180/123	77/95	225/110	130/50	702/458

*p < 0.05, **p < 0.01 for the between-treatment difference in the incidence of transitioning.

To evaluate the impact of potential baseline covariates other than the baseline category (age, race, current depression status, and previous antidepressant use) on the probability of different outcome category membership, a stepwise variable selection was performed within each treatment group using the same multinomial logistic regression model (see statistical methods section). Overall, after adjusting for multiplicity, none of the available baseline predictors was seen to substantially improve the prediction model, once the baseline category was included in the model. As weak evidence, we report that within the duloxetine group, “previous antidepressant use” appeared to decrease the likelihood of the worst outcome compared to all other categories (overall p = 0.046).

### Long-term Effect of Duloxetine

As a sensitivity analysis to assess the changes over a longer period of time, the descriptive rules were applied on the open-label long-term safety study in which patients received duloxetine for up to 62 weeks [20]. Table 3 summarizes the frequencies for switching categories from Week 14 to Week 26. The vast majority of patients either maintained or improved their category; even in the “best” category, 74.3% of patients maintained their status at Week 26 compared to Week 14.

**Table 3:** **Relative frequencies for transitioning from week 14 to week 26 in duloxetine patients of a long-term safety study**

	Percentage of duloxetine patients					
	Category at week 26					
Category at Week 14	1 (worst)	2 (physically poor)	3 (mentally poor)	4 (moderate)	5 (best)	Total number of patients
1 (worst)	43.6	18.0	23.1	12.8	2.6	39
2 (physically poor)	7.8	28.1	25.0	28.1	10.9	64
3 (mentally poor)	18.4	15.8	47.4	10.5	7.9	38
4 (moderate)	0	23.7	11.9	37.3	27.1	59
5 (best)	5.7	8.6	2.9	8.6	74.3	35
Total number of patients	31	48	51	52	53	235

A similar analysis was performed on 2 of the 4 studies used in the cluster analysis that included an extension phase. Again, more than 70% of patients who had reached the best category at Week 12/13 (depending on the study) were able to maintain their good health condition and thus stay in Category 5 at Week 27/28, after another 15 weeks of duloxetine treatment.

## Discussion

Since FM is a complex and heterogeneous condition, the European League against Rheumatism (EULAR) recommends a comprehensive assessment of pain, function, and psychosocial context [36]. One main goal of the pooled analysis presented here was to support the validity of distinct subgroups of FM patients suggested by previous cross-sectional studies. We also explored the usefulness of these subgroups for the clinician in positioning the FM patient along the spectrum of different symptom and functioning domains and in predicting outcome and differential response to treatment, which may provide guidance on the level and type of care and support for the patient’s needs.

One major difference between this analysis and previous studies of FM patients [22, 37, 38] is that the clustering was not based on patient characteristics at baseline but on a wide range of post-baseline efficacy measures collected after 12 weeks of treatment with duloxetine. This allowed the definition of the “best” Cluster 5 that included patients with low pain levels and nearly normal mental and physical status.

Cluster 5 reflects a pronounced positive development over time such that patients in this cluster seem to be mostly recovered from FM. Clusters 1 to 4 correspond to some extent with the 3 subgroups of FM patients described by Giesecke et al. [37]. Giesecke identified a group of patients with a high severity of pain combined with a high degree of mental impairment, which would correspond to Cluster 1 (worst) in the present analysis, and a group with a moderate degree of these symptom domains, similar to Cluster 4 (moderate). The third, smaller group identified by Giesecke et al. included patients with a low degree of mood disturbances but with a high tenderness on evoked pain testing, which might overlap to some extent with our Cluster 2 (physically poor). Our cluster analysis results differentiated between 2 distinct symptom domains characterized by either physical impairment (Cluster 2) or mental impairment (Cluster 3), while these 2 resulting clusters were otherwise similar in terms of pain intensity. Similarly, based on 3 symptom factor scores (musculoskeletal, non-musculoskeletal, and cognitive/psychological symptoms), Wilson et al. identified 4 clusters of FM patients where Groups 2 and 3 differ with regard to more pronounced physical and cognitive/psychological symptoms, respectively, similar to our Clusters 2 and 3 [22]. De Souza et al. emphasize anxiety and depression as a differentiating factor, based on a 2-cluster model of the FIQ items [38]. This was confirmed in the present cluster analysis, which differentiated between mental and pain/physical complaints and impairments in patients situated between the worst and moderate levels of overall symptom severity. The comparable results of these other studies and the present findings seem to add validity to the concept of distinct homogenous subgroups within the broad range of FM patients, despite the fact that underlying outcome measures varied between studies and a different construct, based on post-baseline efficacy data, was applied in the present cluster analysis.

The heterogeneity across the clusters also allowed us to establish patient categories that approximate the clusters with high sensitivity and specificity, using distinct descriptive rules based on only 3 key outcome variables (BPI pain interference; FIQ items 14 [interference with work] and 20 [depression]). Using such rules, any given patient can be classified into one of these categories, as long as data on the 3 outcome variables are available. In the present analysis, pain impact severity seemed to be the most important parameter driving classification of a patient into the 2 best categories or the 3 worst categories. For patients with moderate pain interference, the level of depression was decisive for Category 2 versus 3. For patients with low pain interference, “interference with work” determined the split between Category 4 versus 5. These findings may help guide the clinician’s treatment strategy for the individual FM patient.

The SDS has been used as a validated measure of functional improvement and remission for patients suffering from major depression and generalized anxiety disorders [39]. In the present study, the SDS-based remission criterion discriminated very well between clusters with poor outcome (Clusters 1–3) and those with better outcome (Clusters 4–5), but provided little discrimination among Clusters 1–3 (Table 1). Moreover, 91% of patients in Cluster 5 achieved a functional remission based on the SDS criterion, highlighting its utility in patients with FM.

Duloxetine and placebo were compared based on category frequencies at baseline and after 12 weeks of treatment, confirming the results seen in the individual studies [4, 5, 16, 19]. While frequencies per category were similar between treatment groups at baseline, more duloxetine than placebo patients fell into the 2 best categories after 12 weeks. This was confirmed in the analysis of transition probabilities.

Mentally poor patients (Cluster 3) were more likely to deteriorate to Cluster 1 than physically poor patients (Cluster 2), suggesting that mental impairment is the more detrimental co-morbidity compared to physical impairment influencing outcome category. Furthermore, a better treatment effect on the change in categories from baseline to Week 12 was observed in physically impaired patients. This supports the independent analgesic properties of duloxetine and suggests that it is a beneficial treatment for FM patients, irrespective of the presence of mental impairment.

Aside from the most predictive factors, i.e. treatment and baseline category, previous use of antidepressants was identified as a modestly significant predictor of outcome category. Results indicated that duloxetine-treated patients with previous antidepressant use were doing moderately better. However, it is not clear if duloxetine is protecting against a history of depression and/or if it is an indication that duloxetine as an SNRI has a positive effect on both pain and emotional problems in FM, in contrast to the most commonly used antidepressants, i.e. selective serotonin reuptake inhibitors (SSRIs). Future studies would be needed to confirm this hypothesis in FM, to support the literature reporting the beneficial role of duloxetine in patients with MDD and painful physical symptoms who were switched from failed initial treatment with SSRIs or other SNRIs [40, 41].

A sustained effect of duloxetine was seen when classification rules were applied to the long-term study. The “best” outcome category at 14 weeks was maintained at 24 weeks in almost 75% of patients. About 64% of patients with “moderate” outcome either maintained their status or improved to the “best” category. Considering that the remainder of patients in this category worsened (36%), the presence of mild residual symptoms may indicate a risk for relapse. On the other hand, more than half of the patients in the “worst” category at Week 14 were able to improve their status with duloxetine treatment up to Week 26.

This cluster analysis confirms earlier findings that FM patients are heterogeneous with different levels of impairment [37, 38], emphasizing the need for treatments tailored to the individual patient while also taking into account both the mental and physical symptoms of the patient. Some patients with minor impairment could be treated in primary care with advice and pharmacological or non-pharmacological support. The more complex patients with a high level of physical and/or mental impairment are better treated in tertiary care with a tailored multidisciplinary approach, as also suggested by Van Koulil [42, 43]. According to the individual patient’s co-morbidity, such treatment should not only focus on pain medication, but also on education, treatment of secondary depressive/anxiety disorders, functional rehabilitation, and psychotherapy. A meta-analysis by Häuser provided strong evidence that multi-component therapy has beneficial short-term effects on the main symptoms of FM, but has no effect in the long term [44]. Häuser therefore underlined the need to develop strategies to maintain a long-term benefit. This process may need to focus on teaching self-management strategies and addressing perpetuating personality factors such as perfectionism, and on treating mental impairment, as this seems to be one of the more negative prognostic factors.

Limitations of the described analyses and results were as follows:

- Data were from registration studies. Study patients were selected based on the respective trial’s entry criteria, and variables were collected only in relation to the studies’ objectives. The presented results may therefore not be generalizable and representative for the general FM patient population.
- The analysis included female patients only.
- An LOCF approach was applied to substitute missing values from withdrawals, which may have exaggerated poorer outcome (patients in Clusters 1, 2, and 3) because the LOCF methodology ignores the fact that patients may have improved if they stayed on treatment for a longer period of time or until the study end. We conducted some additional sensitivity analyses that demonstrated that when LOCF outcomes are replaced with outcomes imputed from repeated measures likelihood based modeling (data not shown here), then patients with poorer outcomes who discontinued early would achieve somewhat better outcomes. However, qualitatively, clusters were similar to those obtained with LOCF imputation.
- The duloxetine doses administered were not consistent and varied depending on the source study (30 mg QD or BID, 60 mg QD or BID, or 120 mg QD). With the general idea to assess rather broadly the difference between placebo and duloxetine in forming different outcome clusters different doses had not been taken into account.
- Due to the limited availability of scales, other domains such as personality traits or tenderness could not be assessed.

## Conclusions

These analyses show that FM patients are heterogeneous and can be classified into distinct subgroups based on overall symptom and impairment severity, and on the basis of the predominance of mental versus physical symptoms for patients with intermediate overall severity level. Treatment with duloxetine showed higher improvement rates than with placebo, and the majority of patients on duloxetine in any category either maintained or improved their health status beyond 12 weeks. The results underline the need for further research into FM patient subgroups and the importance of a comprehensive patient assessment as a first step towards a tailored treatment.

## Authors’ original submitted files for images

Below are the links to the authors’ original submitted files for images. Authors’ original file for figure 1 Authors’ original file for figure 2 Authors’ original file for figure 3 Authors’ original file for figure 4
