# Supplementary figures and images for: Typology of patients with fibromyalgia: cluster analysis of duloxetine study patients
Source: BMC Musculoskelet Disord. 2014 Dec 23;15:450. doi: 10.1186/1471-2474-15-450 (PMC4364643; doi:10.1186/1471-2474-15-450)

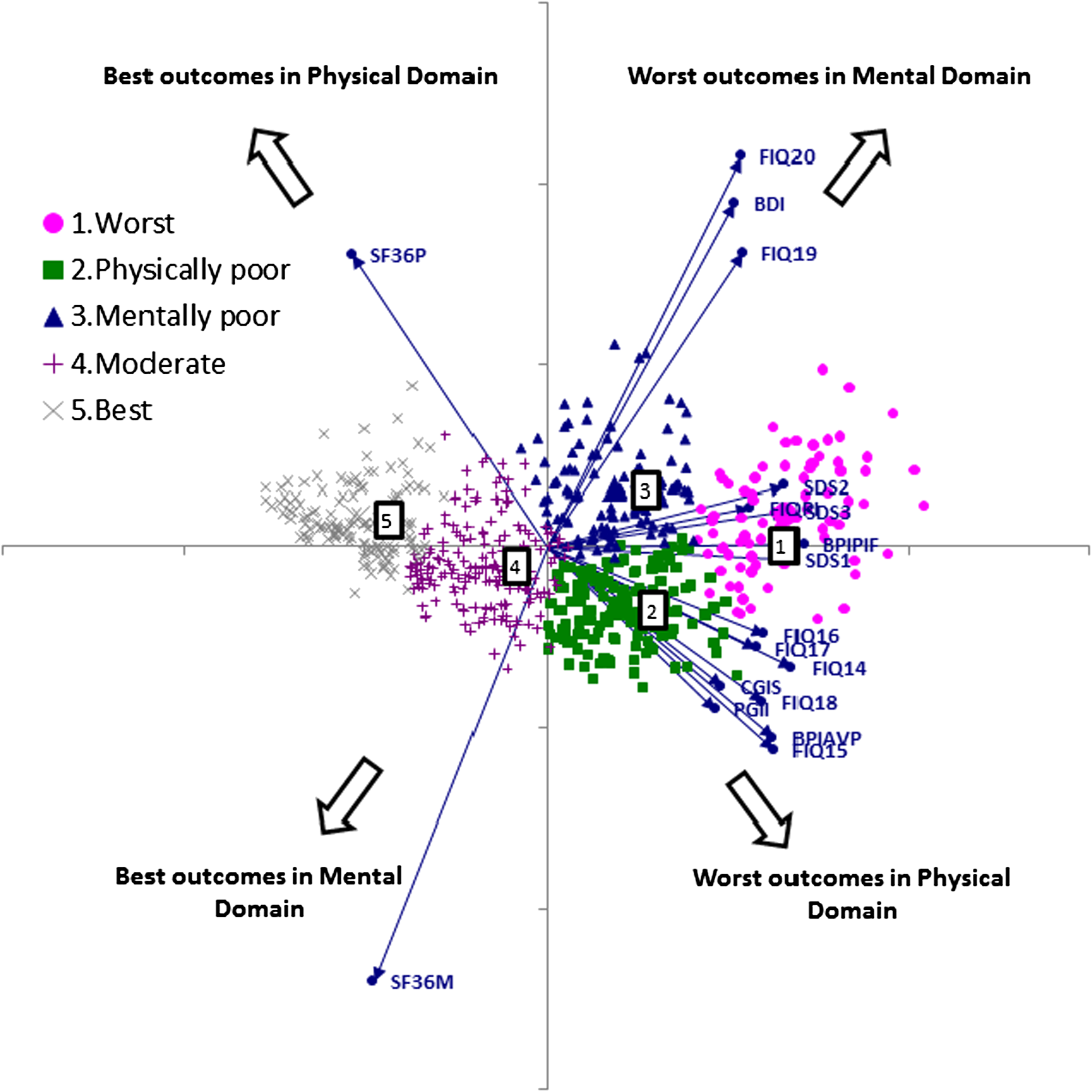

Supplement: Supplementary file 1 — Authors’ original file for figure 1 [file 12891_2014_2393_MOESM1_ESM.tiff]

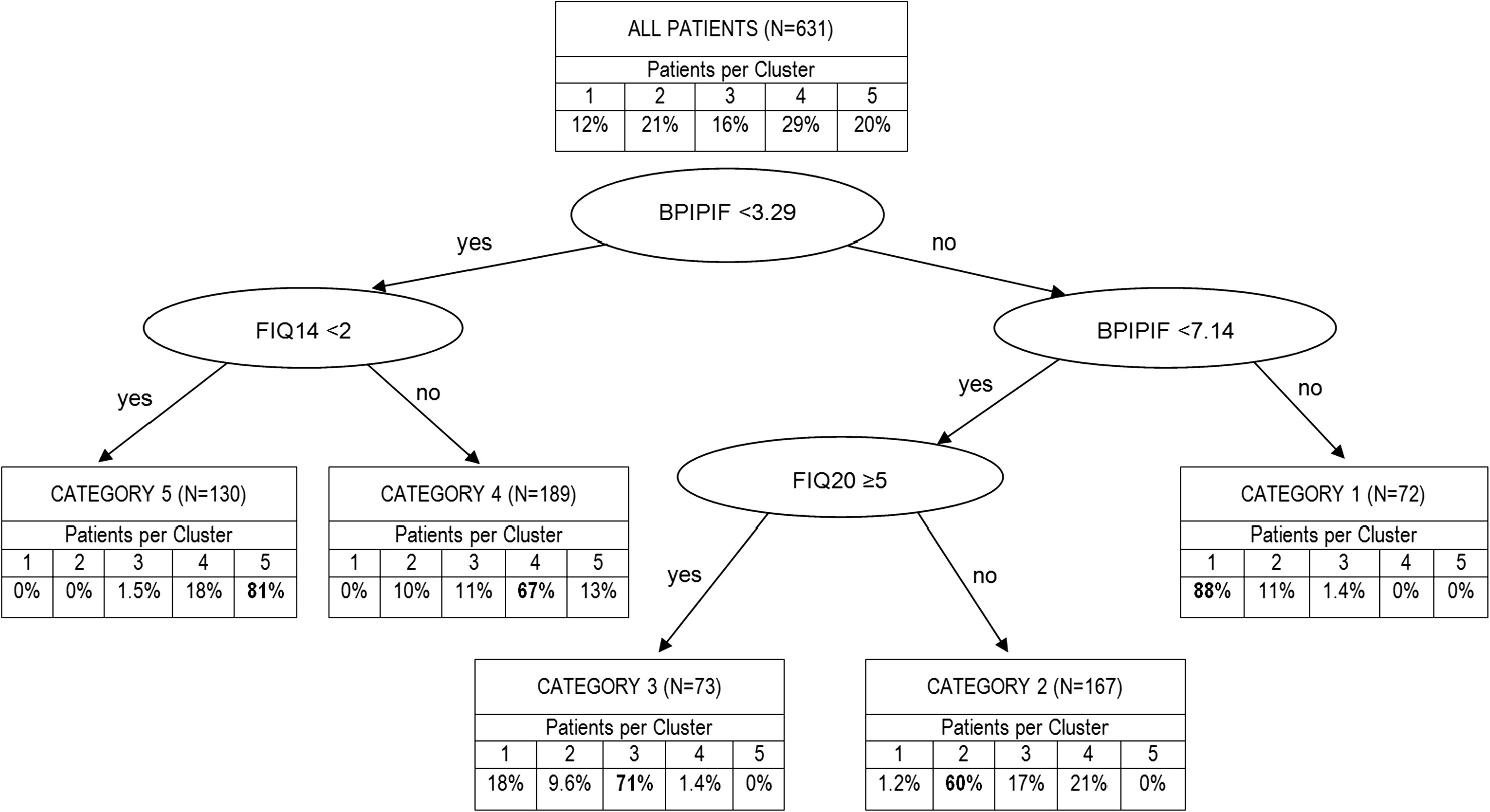

Supplement: Supplementary file 2 — Authors’ original file for figure 2 [file 12891_2014_2393_MOESM2_ESM.tiff]

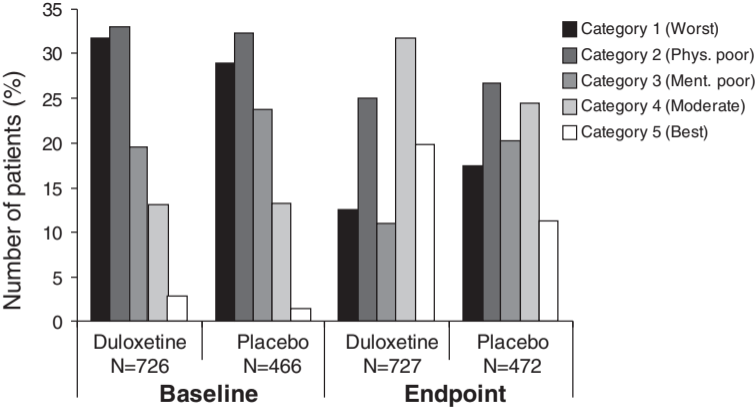

N = number of evaluable patients at baseline and endpoint (Week 2-12, LOCF)

Supplement: Supplementary file 3 — Authors’ original file for figure 3 [file 12891_2014_2393_MOESM3_ESM.pdf]

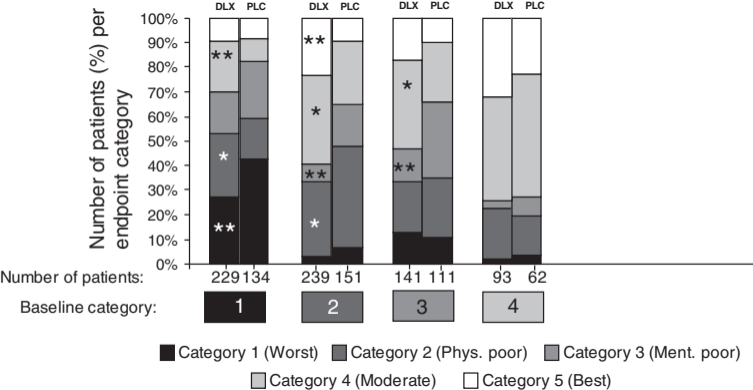

Supplement: Supplementary file 4 — Authors’ original file for figure 4 [file 12891_2014_2393_MOESM4_ESM.pdf]
